# Associations of circulating plasma microRNAs with age, body mass index and sex in a population-based study

**DOI:** 10.1186/s12920-015-0136-7

**Published:** 2015-10-14

**Authors:** Sabine Ameling, Tim Kacprowski, Ravi Kumar Chilukoti, Carolin Malsch, Volkmar Liebscher, Karsten Suhre, Maik Pietzner, Nele Friedrich, Georg Homuth, Elke Hammer, Uwe Völker

**Affiliations:** Department of Functional Genomics, Interfaculty Institute for Genetics and Functional Genomics, University Medicine Greifswald, , Friedrich-Ludwig-Jahn-Str. 15A, D-17475 Greifswald, Germany; Institute of Mathematics and Informatics, Ernst-Moritz-Arndt-University, Greifswald, Germany; Department of Physiology and Biophysics, Weill Cornell Medical College in Qatar, Education City, PO Box 24144, Doha, Qatar; Helmholtz Zentrum München, Germany, Research Centre for Environmental Health, Neuherberg, Germany; Institute of Clinical Chemistry and Laboratory Medicine, University Medicine Greifswald, Greifswald, Germany; DZHK (German Centre for Cardiovascular Research), partner site Greifswald, Greifswald, Germany

**Keywords:** BMI, Age, Sex, Circulating microRNA, miRNA, Association studies, Plasma, Blood

## Abstract

**Background:**

Non-cellular blood circulating microRNAs (plasma miRNAs) represent a promising source for the development of prognostic and diagnostic tools owing to their minimally invasive sampling, high stability, and simple quantification by standard techniques such as RT-*q*PCR. So far, the majority of association studies involving plasma miRNAs were disease-specific case-control analyses. In contrast, in the present study, plasma miRNAs were analysed in a sample of 372 individuals from a population-based cohort study, the Study of Health in Pomerania (SHIP).

**Methods:**

Quantification of miRNA levels was performed by RT-*q*PCR using the Exiqon Serum/Plasma Focus microRNA PCR Panel V3.M covering 179 different miRNAs. Of these, 155 were included in our analyses after quality-control. Associations between plasma miRNAs and the phenotypes age, body mass index (BMI), and sex were assessed *via* a two-step linear regression approach per miRNA. The first step regressed out the technical parameters and the second step determined the remaining associations between the respective plasma miRNA and the phenotypes of interest.

**Results:**

After regressing out technical parameters and adjusting for the respective other two phenotypes, 7, 15, and 35 plasma miRNAs were significantly (*q* < 0.05) associated with age, BMI, and sex, respectively. Additional adjustment for the blood cell parameters identified 12 and 19 miRNAs to be significantly associated with age and BMI, respectively. Most of the BMI-associated miRNAs likely originate from liver. Sex-associated differences in miRNA levels were largely determined by differences in blood cell parameters. Thus, only 7 as compared to originally 35 sex-associated miRNAs displayed sex-specific differences after adjustment for blood cell parameters.

**Conclusions:**

These findings emphasize that circulating miRNAs are strongly impacted by age, BMI, and sex. Hence, these parameters should be considered as covariates in association studies based on plasma miRNA levels. The established experimental and computational workflow can now be used in future screening studies to determine associations of plasma miRNAs with defined disease phenotypes.

**Electronic supplementary material:**

The online version of this article (doi:10.1186/s12920-015-0136-7) contains supplementary material, which is available to authorized users.

## Background

MicroRNAs (miRNAs) are small ~22 nt long non-coding RNAs which play important regulatory roles by targeting mRNAs for degradation or mediating translational repression. Thus, they affect a wide range of physiological and pathophysiological processes including cell differentiation, proliferation, apoptosis, angiogenesis or inflammation [1, 2]. Besides their common intracellular localization, miRNAs are present in different body fluids, particularly in blood. The factors that determine the levels of extracellular miRNAs, e.g. in plasma, such as active secretion or passive release due to cell lysis as well as the functional roles of these plasma miRNAs are still under investigation. Non-cellular blood circulating microRNAs (in the remainder of this paper referred to as plasma miRNAs) are highly ribonuclease-resistant because they are either enclosed in membranous vesicles as apoptotic bodies and exosomes or localized in complexes with RNA-binding proteins (Ago2), high-density lipoproteins or nucleophosmin [3–5]. Recent studies have proposed a hormone-like role for circulating miRNAs in intercellular communication [3, 4]. The putative value of plasma miRNAs as predictive and diagnostic biomarkers motivated the recent large-scale profiling of these molecules in the context of many diseases, such as cancer, diabetes, multiple sclerosis, coronary artery disease or myocardial infarction [6–8]. A selection of 19 potentially informative plasma miRNAs was investigated by Zampetaki et al. [8] in a prospective, population-based study on cardiovascular disease. The study revealed associations between several miRNAs and the incidence of myocardial infarction and highlighted platelets as a major source for the plasma miRNA pool [8]. Few other population based studies were published on circulating miRNAs derived from blood cells [9] or serum [10]. Usually, the screening for candidate miRNAs has so far been performed on pooled or individual samples of cases and controls. Selected miRNAs have been measured by RT-*q*PCR in the entire sample or a sub-sample of the study cohort. Selection bias and co-morbidities might have affected the phenotype under investigation [11]. For instance, studies on miRNA levels detected in human plasma samples provided evidence for age-associated miRNA levels in blood [12, 13]. Such associations are almost certainly relevant in studies on age-related diseases such as cancer or cardiovascular disorders. Recently, the impact of age and sex on circulating miRNA was explored in peripheral blood using microarray technology [14]. This study highlighted the importance of age matching case-control studies whereas sex seemed to have a less pronounced effect on the miRNA levels [14]. The use of matched case-control cohorts or adjustment for age and sex is therefore warranted [11].

In the present study, we investigated plasma miRNAs prepared from human plasma using a reverse transcription-quantitative PCR (RT-*q*PCR) approach based on the Serum/Plasma Focus microRNA PCR Panel V3.M (Exiqon) which provides a higher sensitivity and specificity compared to array-based techniques [15]. We assessed the association of age, body mass index (BMI), and sex with miRNA levels in a sample of 372 individuals from the population based Study of Health in Pomerania (SHIP-TREND) applying stringent data pre-processing and taking into account technical as well as blood cell parameters [16].

## Methods

### Ethics statement

The study has been conducted according to the recommendations of the Declaration of Helsinki. The study protocol of SHIP was approved by the medical ethics committee of the University of Greifswald. Written informed consent was obtained from each of the study participants.

### Study design

Initially, EDTA-plasma samples from 384 participants were randomly selected from the SHIP-TREND cohort. Study design and sampling methods for SHIP-TREND were previously described [16]. Briefly, SHIP-TREND is a longitudinal population-based cohort study assessing the prevalence and incidence of common diseases and their risk factors. Study participants were randomly selected from the population registries in North- and East-Pomerania.

The laboratory workflow for RT-*q*PCR based miRNA analysis using the Serum/Plasma Focus microRNA PCR Panel V3.M (Exiqon A/S, Vedbaek, Denmark) involved several quality control steps which resulted in the exclusion of 12 plasma samples. The descriptive statistics of the remaining study participants are provided in Table 1. Blood cell parameters were measured on an automated haematology system (XT 200 or XE 5000, Sysmex, Europe).

**Table 1:** Characterization of the SHIP-TREND sample

	Sex	Age (years)		BMI (kg/m^2^)	
	number	Mean ± SD	range	Mean ± SD	range
male	187	49.3 ± 14.5	22-79	27.6 ± 3.9	17.7-39.0
female	185	50.2 ± 13	22-79	27.4 ± 5.0	18.7-48.1
total	372	49.7 ± 13.8	22-79	27.5 ± 4.4	17.7-48.1

### Preparation of plasma miRNAs

Non-cellular blood circulating miRNAs were isolated from 200 μl plasma using the miRCURY™ RNA Isolation Kit –Biofluids (Exiqon A/S) according to the manufacturer’s instructions. To ensure high and reproducible RNA yield from EDTA-plasma samples, bacteriophage MS2 carrier RNA was added to each sample during the purification procedure. Reverse transcription reactions were performed using Universal cDNA Synthesis Kit II (Exiqon A/S) according to the manufacturer’s instructions**.** Before using RNA samples for miRNA profiling, the yield of typical plasma miRNAs, absence of PCR inhibitors as well as of haemolysis in the samples was assessed by use of a microRNA QC PCR Panel (Exiqon A/S).

### Profiling of plasma miRNAs

Exiqon Serum/Plasma Focus microRNA PCR Panels, 384 well (V3.M) were used in a RT-*q*PCR approach to determine the plasma levels of 179 human miRNAs. The *q*PCR was performed using a 7900 HT Real-time PCR system (Applied Biosystems, Carlsbad, CA, USA) with 42 amplification cycles employing the cycling parameters recommended by Exiqon. Raw data were processed using SDS 2.4 (Applied Biosystems) to assign the baseline and threshold for Ct (threshold cycle: the PCR cycle at which the target is quantified in a given sample, according to Real-time PCR Data Markup Language (RDML) guidelines [17]) determination. To determine the technical variation between the Exiqon Serum/Plasma Focus microRNA PCR Panel plates, the inter-plate calibrator (IPC) (UniSp3) was analysed. Ct values of the IPC were 20 ± 0.2 (mean ± SD) across all samples, and thus highly similar.

### Computational analyses

All computational analyses were implemented in R (3.1.2 “Pumpkin Helmet”) [18]. The code is available upon request. Generally, a miRNA-wise two-step regression procedure was employed. Through the evaluation of a first regression model the data were corrected for technical influences (see Regressing-out of technical parameters). The second step comprised the assessment of associations between miRNAs and phenotypes in a separate regression model (see Analysis of associations between miRNA levels and phenotypes).

### Data pre-processing

We ran the RT-*q*PCR for 42 cycles and specified the detection cut off according to manufacturer’s recommendation [15]. The Ct value threshold established in [15] for the very sensitive Exiqon platform has already been successfully employed in other studies on low-abundant miRNAs [19, 20]. Thus, also in our study Ct values below 37 were considered for quantification and Ct values above 37 were treated as missing because they were considered to be too close to the detection limit of the assay. Remaining Ct values were normalized to the lower quartile per sample. That is, per sample the lower quartile across all Ct values, excluding spike-ins, was subtracted from each individual sample Ct value, yielding ∆Ct values (small ∆Ct values indicate high miRNA levels). Only miRNAs with at least 100 valid (i.e. non-missing) ∆Ct values and normal ∆Ct value distribution across all samples were kept. Thus, all association analyses are based on a set of 155 miRNAs that satisfied these criteria.

### Regressing-out of technical parameters

Plasma miRNA data were influenced by a number of technical parameters. Among these were the storage time of plasma samples in the biobank ranging from 1035 to 1774 days (dt_biobank) and the Ct values of synthetic spiked-in miRNAs monitoring the efficiency of miRNA extraction (UniSp2 and the difference between Ct values of UniSp4 and UniSp2). Before further analyses, these parameters were regressed out of the data. To that end, linear regression according to the model ∆Ct ~ dt_biobank + UniSp2 + UniSp4-UniSp2 , which treats the ∆Ct values as dependent variables and the technical parameters as independent variables was performed for each miRNA. The resulting residuals represent the variance in miRNA levels that cannot be explained by the aforementioned technical parameters. These residuals were used as dependent variables in later models to detect associations between miRNAs and phenotypes.

### Analysis of associations between miRNA levels and phenotypes

To identify associations between plasma miRNA levels and the phenotypes investigated in this study, linear regression models were fitted for each miRNA separately. These models contained the residuals of the model for technical adjustment (see Regressing-out of technical parameters) as dependent variables. That is, the goal of these models was to explain the variance in miRNA levels not due to technical parameters. As independent variables the phenotype(s) examined or adjusted for (i.e. age, BMI, sex), as well as important blood cell parameters (BCPs) (see Identification of blood cell parameters) were used. The strength of a miRNA-phenotype association was assessed by the Benjamini-Hochberg-corrected *p*-value (*q-*value) of the corresponding model coefficient.

### Identification of blood cell parameters

An elastic net regularized regression model [21] was employed to identify important BCPs that affect miRNA levels. The motivation to identify BCPs via an elastic net was two-fold. First, the elastic net selects the smallest subset of independent variables most accurately predicting the dependant variable. This results in the incorporation of as few BCPs into our models as possible, while accounting for as much variation in the miRNA data as possible. Second, the elastic net accounts for correlation between covariates. This is important here, since many BCPs are inter-correlated. The elastic-net regularized linear regression models were evaluated through a leave-one-out cross-validation as implemented in the R package glmnet (v 1.9-8) [22]. These models comprised ∆Ct values as dependent variables and technical parameters and BCPs as independent variables (for a list of all BCPs, see Additional file 1: Table S1). Alpha for these models was set to 0.5 to balance-out the L1 and L2 penalties. BCPs frequently selected by the elastic net across all miRNAs were incorporated into linear regression models assessing miRNA – phenotype associations. These BCPs were haematocrit, platelet count, and mean platelet volume. A Manhattan plot of these BCPs in the linear model is provided as Additional file 1: Figure S1.

## Results

For the present study, data from 187 men and 185 women were used. Age of the participants varied between 22 and 79 years and the body mass index varied between 17.7 and 48.1 (kg/m^2^) (Table 1).

### Association of plasma miRNA levels with age, BMI, and sex

**Fig. 1:**
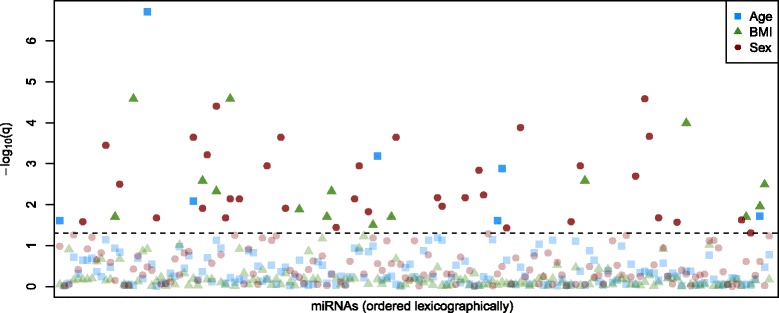
Association *q*-values of miRNAs in two-step regression models with adjustment for technical and biological parameters. The -log_10_(*q*) values of the linear regression analysis of miRNA levels and phenotypes age (blue rectangle), BMI (green triangle) and sex (red circle) are depicted. *Q*-values were obtained via Benjamini-Hochberg (BH) multiple testing correction of raw *p*-values. The dotted line marks the significance threshold of *q* = 0.05. Plasma miRNAs are lexicographically arranged on the x-axis (though not labelled individually)

For the present proof-of-principle study, we investigated associations between plasma miRNA levels and the phenotypes BMI, sex, and age (Fig. 1).

**Fig. 2:**
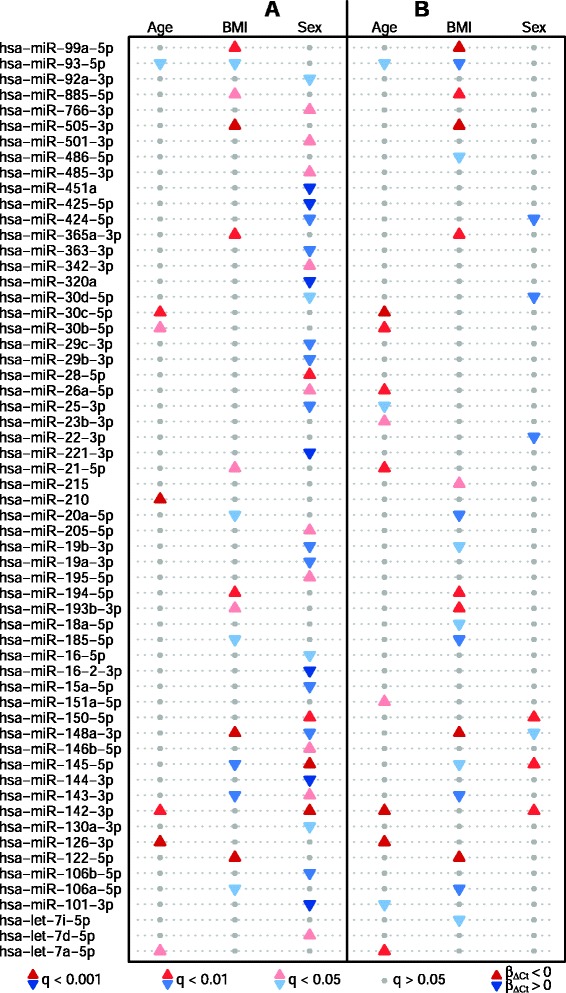
Associations of miRNAs and direction of effect. The effect direction is shown for each miRNA significantly associated with age, BMI and sex after adjustment for **a**) technical covariates and the respective other two phenotypes and **b**) after additional adjustment for blood composition. Each row represents a miRNA and each column shows the association with a specific phenotype. The magnitude of the Benjamini-Hochberg corrected *p*-values (*q*-values) is indicated by the colour tone. Darker colour indicates a lower *q*-value. Positive correlations of miRNA levels (β_∆Ct_ < 0, since smaller ∆Ct values indicate higher miRNA levels) are indicated by red upward triangles while negative correlations (β_∆Ct_ > 0, since larger ∆Ct values indicate lower miRNA levels) are indicated by blue downward triangles. Sex has been numerically encoded as the number of X-chromosomes. Hence, positive correlation here indicates a female-specific miRNA while negative correlation indicates a male-specific miRNA. A grey dot indicates no significant association

The linear regression models incorporated all three phenotypes. Significant (*q* < 0.05) associations with age were detected for seven miRNAs. The strongest were observed for *hsa*-miR-126-3p and *hsa*-miR-21-5p (*q* < 0.001) (Fig. 2a, and Additional file 2: Table S2). Altogether 15 miRNAs were significantly associated with BMI. Here, the most significant associations were observed for *hsa*-miR-122-5p, *hsa*-miR-148a-3p and *hsa*-miR-505-3p (*q* < 0.001) (Fig. 2a and Additional file 3: Table S3). Regarding sex, 35 miRNAs were found to be significantly associated (Fig. 2a and Additional file 4: Table S4). Among these, several miRNAs such as *hsa*-miR-145-5p, *hsa*-miR-451a, *hsa*-miR-143-3p, *hsa*-miR-16-2-3p are known to originate from blood cells and to be involved in haematopoiesis.

**Fig. 3:**
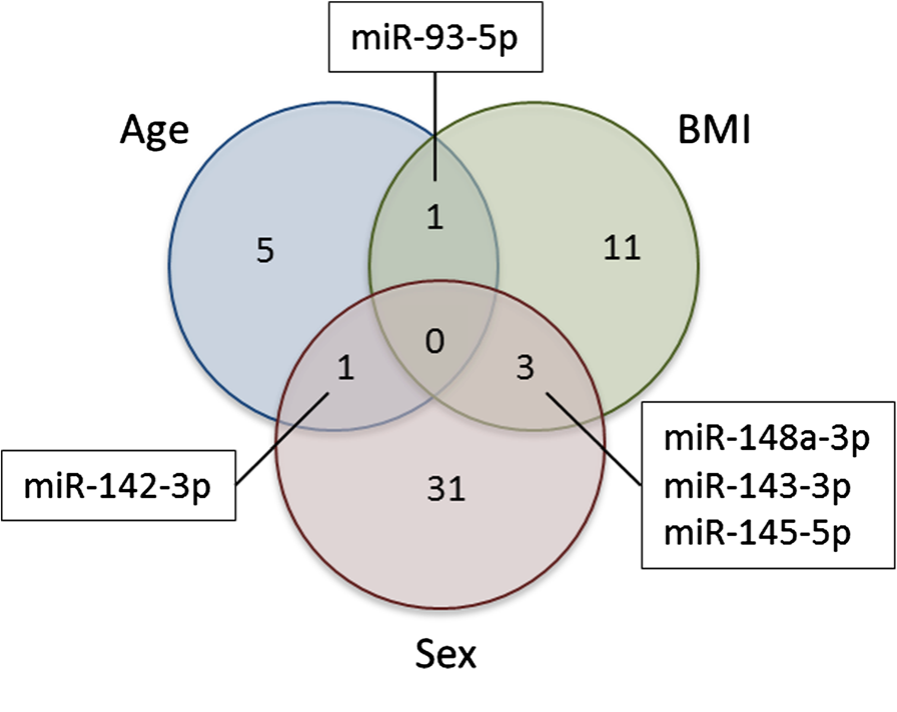
Overlap of associations of miRNAs for different phenotypes. Venn diagram of miRNAs significantly (*q* < 0.05) associated with the three phenotypes age, BMI, and sex, in two-step regression models incorporating technical and biological parameters

The number of miRNAs associated to multiple phenotypes was strikingly small (Fig. 3). Only *hsa*-miR-93-5p was associated with both age and BMI. Also the overlap between age and sex was restricted to only one miRNA, namely *hsa*-miR-142-3p. The miRNAs *hsa*-miR-143-3p, *hsa*-145-5p, and *hsa*- miR-148a-3p were associated with both sex and BMI. There was no miRNA associated with all three phenotypes.

### Modified associations after adjustment for blood cell parameters

Additional adjustment was performed using appropriate blood cell parameters (BCPs, see Methods section for details). The number of significantly (*q* < 0.05) age-associated miRNAs rose to 12 after adjustment for sex, BMI, and BCPs, 7 of which overlapped with the previous results (Fig. 2b, Additional file 2: Table S2). The strongest associations with age were observed for *hsa*-miR-126-3p, *hsa*-miR-30c-5p, and *hsa*-miR-142-3p (*q* < 0.001) (Fig. 2b, Additional file 2: Table S2). However, the effect sizes (β_∆Ct_ from linear regression) of associations with age were quite small (0.01 to -0.01). Adjustment for age, sex, and BCPs resulted in the identification of 19 significantly BMI-associated miRNAs, 15 of which overlapped with the previous results (Fig. 2b, Additional file 3: Table S3). The most significant associations with BMI were still observed for *hsa*-miR-122-5p, *hsa*-miR-148a-3p and *hsa*-miR-505-3p (*q* < 0.001).

Adjustment for age, BMI, and BCPs reduced the number of significantly sex-associated miRNAs from 36 to 7 (Fig. 2b, Additional file 4: Table S4). The association strength of the selected BCPs (haematocrit, platelet count, and mean platelet volume) is shown in the Manhattan plot in Additional file 1: Figure S1. Nevertheless, remaining associations were still accompanied by profound effect sizes (β_∆Ct_ = 0.33 to –0.35). The most significant associations with sex were observed for *hsa*-miR-145-5p, *hsa*-miR-150-5p and *hsa*-miR-142-3p (*q* < 0.01).

**Fig. 4:**
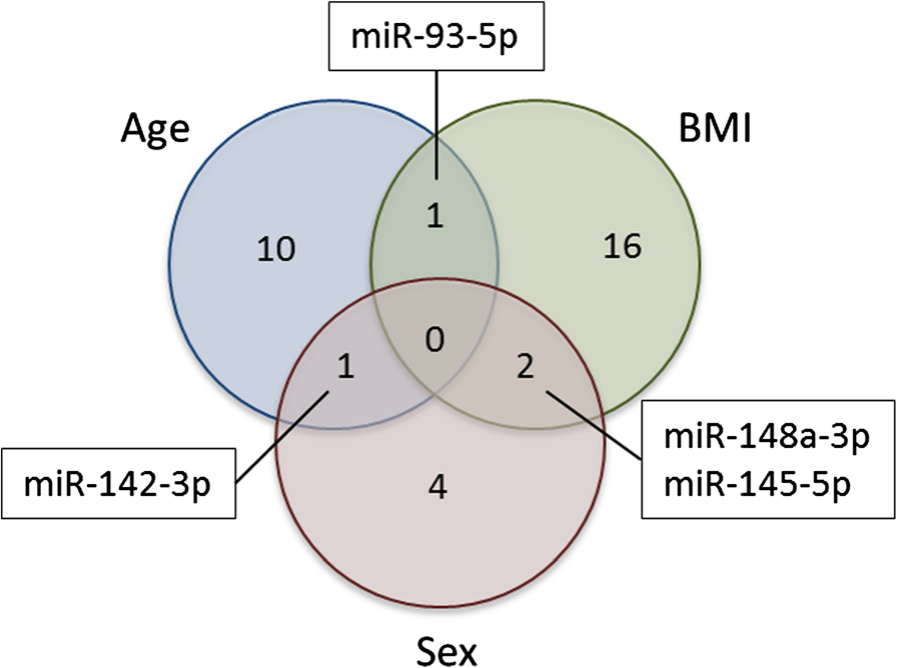
Overlap of associations of miRNAs for different phenotypes after adjustment for blood cell parameters. Overlap of miRNAs significantly (*q* < 0.05) associated with the three phenotypes age, BMI, and sex in two-step regression models incorporating technical parameters, all three phenotypes, and blood cell parameters

The overlap of association of miRNAs with different phenotypes after the adjustment for the respective other two phenotypes and BCPs was also investigated. In the fully adjusted models, only few miRNAs were significantly associated with more than one phenotype (Fig. 4): *hsa*-miR-93-5p with both BMI and age, *hsa*-miR-148a-3p and *hsa*-miR-145-5p with both BMI and sex, and *hsa*-miR-142-3p with both age and sex. Again, none of the miRNAs was associated with all three phenotypes.

## Discussion

In the last few years, the interest in non-cellular blood circulating miRNAs (plasma miRNAs) as biomarkers present in easily accessible body fluids increased continuously. Various studies analysed associations between plasma miRNAs and specific disease phenotypes, mostly using a case-control design [11]. However, these studies were frequently limited by relatively small sample sizes as well as a number of biases, e.g. the lack of appropriate adjustment for confounding factors such as age [11]. So far a limited number of large population-based phenotype-miRNA association studies have been performed [8–11]. Furthermore, while whole blood is indeed easily accessible for sampling, its composition is complex. Different cell types and tissues in contact with blood might contribute miRNA species to the plasma miRNA pool in different proportions. Without appropriate adjustment, plasma miRNA profiles will always partially reflect the individual blood composition at the sampling time [23, 24]. Thus, in the context of the search for highly informative biomarkers, it appears reasonable to focus on miRNAs that are not or only marginally affected by the BCPs.

In the present study, we quantified plasma miRNA levels of 372 individuals from the population-based SHIP-TREND cohort and analysed the associations of these miRNA profiles with age, BMI, and sex under consideration of the BCPs. The numbers of age-associated and BMI-associated miRNAs were similar. A positive age-correlation was found for *hsa*-miR-126-3p, thereby confirming a recently published study [25]. Further miRNAs detected to be associated with age in this study such as *hsa*-miR-30, *hsa*-miR-93, *hsa*-miR-21 and *hsa*-miR-142-3p were also previously reported as age-related [14, 26, 27]. However, *hsa*-miR-17, *hsa*-miR-19b, *hsa*-miR20a and *hsa*-miR-106a could not be validated as age-associated plasma miRNAs in our population based study [28]. Further miRNAs such as *hsa*-miR-26a-5p, *hsa*-let-7a-5p, *hsa*-miR-101-3p and *hsa*-miR-23b-3p were found to be age associated but have not yet been described. However, in all cases, the effect sizes of the age-miRNA-associations were rather small, indicating a less pronounced relationship between age and plasma miRNA levels, as compared to, e.g., BMI-miRNA-associations.

Several plasma miRNAs were significantly associated with BMI in the present study. One of the most prominent was *hsa*-miR-122-5p which is highly abundant in the adult liver [29] where it acts as a key regulator of cholesterol and fatty-acid metabolism [30]. This miRNA was recently described as a serum biomarker for liver injury in chronic hepatitis B and C, non-alcoholic fatty liver disease (NAFLD), and drug-induced liver disease [31–34]. Consistently, the comparison of miRNAs known to be synthesized in large amounts in adult human liver [35] with the significantly BMI-associated plasma miRNAs detected in this study revealed an overlap of 12 miRNAs (*hsa*-miR-99a, *hsa*-miR-194, *hsa*-miR-143, *hsa*-miR-93, *hsa*-miR-185, *hsa*-miR-885, *hsa*-miR-193b, *hsa*-miR-145, *hsa*-miR-19b, *hsa*-miR-18a, *hsa*-miR-486, and *hsa*-miR-148a). The prominent BMI-associated miRNAs belong to different families organized in clusters such as *miR-106b ~ 25* (*hsa*-miR-93), *miR-106a ~ 363* (*hsa*-miR-106a) and *miR-17 ~ 92* (*hsa*-miR-18a, *hsa*-miR-20a and *hsa*-miR-19b-1). The obvious high portion of liver-specific miRNAs whose blood levels were found to be positively correlated with BMI points towards release of these miRNAs from lysing hepatocytes into the circulation as a consequence of subclinical or/ and manifest NAFLD which is in turn strongly positively associated with an increased BMI.

A recently published microarray-based study on the associations between peripheral blood circulating miRNAs with age as well as sex revealed only a limited association between sex and miRNA patterns [14]. In the present study, we also investigated the association between sex and plasma miRNA profile. Before adjustment for BCPs there were more miRNAs significantly associated with sex than with age or BMI. However, a high proportion of these sex-associated miRNAs most probably originates from blood cells. Monocytes, thrombocytes, granulocytes, lymphocytes, reticulocytes and erythrocytes all contain several cell type specific as well as ubiquitously expressed miRNAs in varying amounts [24, 36]. The total blood cell mass in the circulation of women is generally smaller compared to men. This was confirmed for multi-ethnic populations [37] and is reflected in a 12 % lower mean haemoglobin level in female venous blood compared to men [38] as well as in lower haematocrit values. Consistently, in the present study, besides significantly elevated erythrocyte counts in men compared with women (Additional file 1: Table S1), erythrocyte-specific miRNAs such as *hsa*-miR-451a or *hsa*-miR-16-2-3p exhibited higher levels in men, which is also in line with the previously published observation that plasma miRNAs correlate to blood cell counts [24]. As expected, the sex-associated signals for blood-cell-specific miRNAs largely vanished after adjustment for BCPs in the present study.

Regulatory roles in haematopoiesis were described for *hsa*-miR-451 and *hsa*-miR-16. These are involved in the differentiation of erythroid progenitor cells into red blood cells. Similarly, *hsa*-miR-150 activates the differentiation of common lymphoid progenitors into T cells, B cells and natural killer cells [39]. As potential sources of such miRNAs, lymphoid cells such as T cells, B cells (*hsa*-miR-150, *hsa*-miR-142), platelets (*hsa*-miR-142) and monocytes (*hsa*-miR-145) have been mentioned [24, 40]. In our study this is reflected by, e.g. significantly increased platelet levels in women compared to men (Additional file 1: Table S1). Recently, it was hypothesized that differential expression of miRNAs in male and female immune cells contributes to sex differences in immune capabilities and susceptibilities to autoimmune diseases [41].

Hence, in studies associating plasma miRNA levels with specific clinical phenotypes, special attention should be paid to sex differences and BCPs.

It is clear that further increasing the sample size might reveal additional associations that until now did not pass the significance threshold. Furthermore, while the RT-*q*PCR approach based on Exiqon´s Serum/Plasma Focus Panels V3 offers a high specificity and sensitivity on measured miRNAs in a high throughput manner, it is limited with respect to the number of detectable miRNAs [15]. Nevertheless, our results corroborate the general feasibility of association studies with plasma miRNAs.

## Conclusions

In the present association study we demonstrate that plasma miRNA profiles based on a population-based study cohort reflect individual sex, age, and BMI. Therefore, our findings underline the importance of considering these phenotypes as potential covariates in such studies. The established experimental and computational workflow presented here will be used in future screening studies for associations with disease-specific phenotype parameters. Beyond that, replication of our primary association findings in further independent cohorts is intended.

## Additional files

Additional file 1:
**Table S1.** Blood composition parameter. **Figure S1.** *Q*-values of BCPs in the linear regression model. (PDF 182 kb) Additional file 2: Table S2. Significantly age-associated miRNAs in different models. (PDF 187 kb) Additional file 3: Table S3. Significantly BMI-associated miRNAs in different models. (PDF 196 kb) Additional file 4: Table S4. Significantly sex-associated miRNAs in different models. (PDF 227 kb)

## Abbreviations

BCP: Blood cell parameters
BH: Benjamini-Hochberg
BMI: Body mass index
miRNA: microRNA
NAFLD: Non-alcoholic fatty liver disease
RT-*q*PCR: Reverse transcription-quantitative PCR
SHIP-TREND: Study of Health in Pomerania-Trend
