# Supplementary material for: Associations of circulating plasma microRNAs with age, body mass index and sex in a population-based study
Source: BMC Med Genomics. 2015 Oct 14;8:61. doi: 10.1186/s12920-015-0136-7 (PMC4604724; doi:10.1186/s12920-015-0136-7)
Supplement: Additional file 1: — Table S1. Blood composition parameter. Figure S1. Q-values of BCPs in the linear regression model. (PDF 182 kb) [file 12920_2015_136_MOESM1_ESM.pdf]

Additional file 1: Table S1 Blood cell parameters.

	overall						male			female			
Blood cell parameter	$Mean_o$	SD_o	$Median_o$	Min_o	Max_o	N_o	$Mean_m$	SD_m	N_m	$Mean_f$	SD_f	N_f	p(Sex)
Basophiles [%]	0.49	0.30	0.40	0.00	1.90	370	0.50	0.31	186	0.48	0.29	184	7.39E-01
**Eosinophils [%]**	2.65	1.91	2.20	0.10	14.10	370	2.94	1.93	186	2.36	1.86	184	**3.35E-03**
**Haematocrit**	0.42	0.03	0.42	0.28	0.52	370	0.43	0.03	186	0.40	0.03	184	**5.92E-30**
**Haemoglobin [mmol/l]**	8.60	0.76	8.60	5.00	10.70	370	9.05	0.61	186	8.15	0.62	184	**2.46E-36**
Lymphocytes [%]	30.16	7.34	29.90	11.70	50.00	370	29.83	6.85	186	30.48	7.81	184	3.94E-01
**Monocytes [%]**	9.20	2.19	9.10	4.30	16.80	370	9.74	2.27	186	8.65	1.96	184	**1.10E-06**
Neutrophils [%]	57.33	8.31	56.95	31.70	78.70	370	56.82	7.78	186	57.85	8.80	184	2.37E-01
**Platelets [Gpt/l]**	226.80	48.93	220.00	102.00	390.00	370	213.53	43.72	186	240.21	50.36	184	**9.96E-08**
**Erythrocytes [Tpt/l]**	4.65	0.39	4.60	3.50	5.70	370	4.85	0.34	186	4.46	0.32	184	**1.21E-25**
Leukocytes [Gpt/l]	5.71	1.48	5.43	2.80	11.60	370	5.64	1.48	186	5.79	1.47	184	3.45E-01
Mean corpuscular volume [fl]	174.03	43.63	174.00	21.00	282.00	372	171.65	46.05	187	176.43	41.02	185	2.90E-01
**Mean corpuscular haemoglobin [fmol]**	50.42	10.96	50.50	2.00	95.00	372	52.54	10.84	187	48.27	10.69	185	**1.54E-04**
**Mean corpuscular haemoglobin concentration [mmol/l]**	28.65	6.05	28.00	2.00	54.00	372	30.92	5.75	187	26.35	5.47	185	**4.55E-14**
Red blood cell distribution width [%]	20.66	9.89	19.00	5.00	80.00	372	19.72	9.34	187	21.60	10.36	185	6.72E-02
Mean platelet volume [fl]	32.29	30.66	14.00	1.00	79.00	372	35.25	31.51	187	29.30	29.56	185	6.12E-02

SD –standard deviation, N – observations, o-overall, m-male, f-female, p(Sex) – p-value from two-sided t-test for difference of means between sexes

Additional file 1: Figure S1 Q-values of BCPs in the linear regression model.

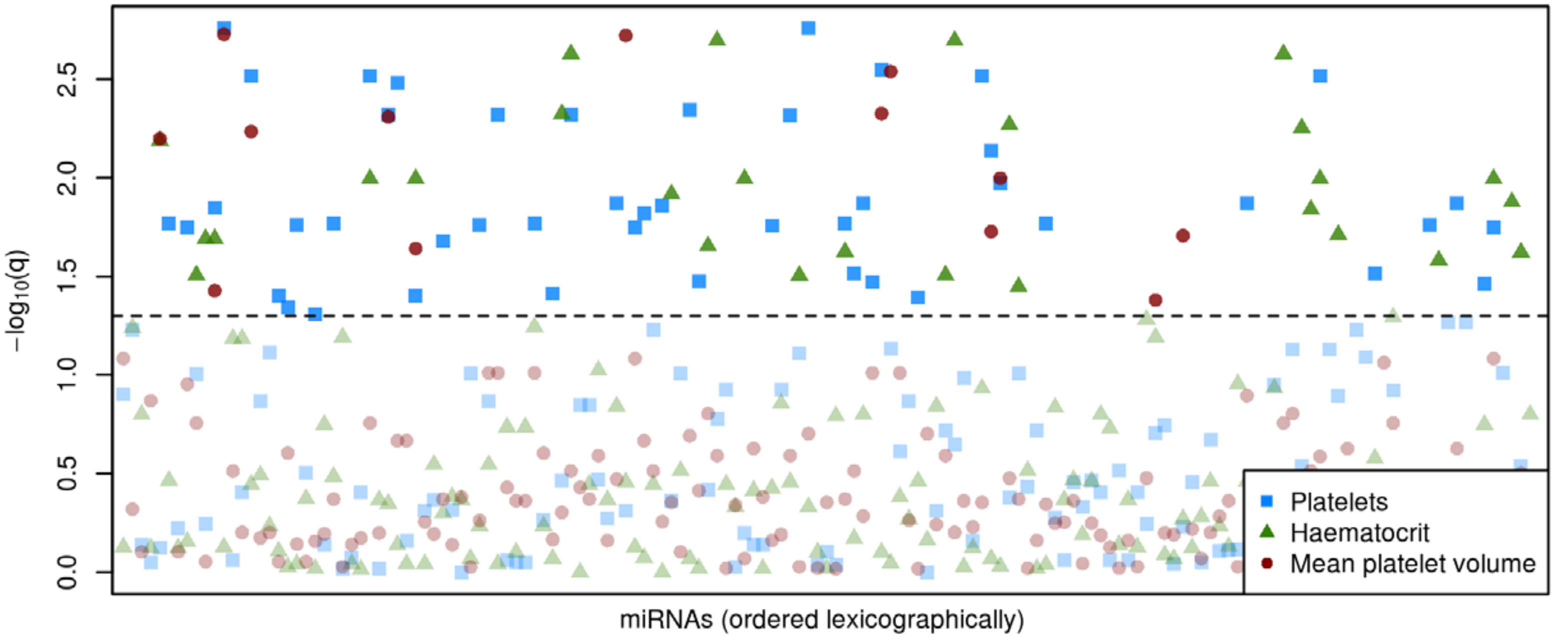

Figure S1: Association *q*-values of BCPs in the linear regression model. The -$\log_{10}(q)$ values of BCPs in the linear regression analysis of miRNA levels and phenotypes. Blue rectangles represent Platelets, green triangles haematocrit, and red circles mean platelet volums. *Q*-values were obtained via Benjamini-Hochberg (BH) multiple testing correction of raw *p*-values. The dotted line marks the significance threshold of q = 0.05. Plasma miRNAs are lexicographically arranged on the x-axis (though not labelled individually).
