# Supplementary material for: Associations of circulating plasma microRNAs with age, body mass index and sex in a population-based study
Source: BMC Med Genomics. 2015 Oct 14;8:61. doi: 10.1186/s12920-015-0136-7 (PMC4604724; doi:10.1186/s12920-015-0136-7)
Supplement: Additional file 2: Table S2. — Significantly age-associated miRNAs in different models. (PDF 187 kb) [file 12920_2015_136_MOESM2_ESM.pdf]

Additional file 2: Table S2. Significantly Age-associated miRNAs in different models.

	Age: adj BMI + sex (7)		Age: adj BMI + sex + BCPs (12)							
miRNA	$\beta_{\Delta Ct}$	*q*	$\beta_{\Delta Ct}$	*q*	N	Accession	gene family	MIPF gene family	Chromo-some	strand
hsa-let-7a-5p	-0.01	2.43E-02	-0.01	8.63E-03	370	MI0000060	let-7	MIPF0000002	chr9	+
hsa-miR-101-3p			0.01	1.97E-02	370	MI0000103	mir-101	MIPF0000046	chr1	-
hsa-miR-126-3p	-0.01	1.93E-07	-0.01	9.70E-09	366	MI0000471	mir-126	MIPF0000115	chr9	+
hsa-miR-142-3p	-0.01	8.10E-03	-0.01	5.03E-04	369	MI0000458	mir-142	MIPF0000084	chr17	-
hsa-miR-21-5p	-0.01	6.46E-04	0.00	2.90E-03	366	MI0000077	mir-21	MIPF0000060	chr17	+
hsa-miR-25-3p			0.01	1.97E-02	370	MI0000082	mir-25	MIPF0000013	chr7	-
hsa-miR-26a-5p			-0.01	9.20E-03	369	MI0000083	mir-26	MIPF0000043	chr3	+
hsa-miR-30b-5p	-0.01	2.43E-02	-0.01	2.90E-03	367	MI0000441	mir-30	MIPF0000005	chr8	-
hsa-miR-30c-5p	-0.01	1.33E-03	-0.01	1.13E-04	368	MI0000254	mir-30	MIPF0000005	chr6	-
hsa-miR-93-5p	0.01	1.89E-02	0.01	1.34E-02	367	MI0000095	mir-17	MIPF0000001	chr7	-
hsa-miR-151a-5p			-0.01	4.05E-02	367	MI0000809	mir-28	MIPF0000057	chr8	-
hsa-miR-23b-3p			-0.01	2.71E-02	368	MI0000439	mir-23	MIPF0000027	chr9	+

Age-associated miRNAs in different linear regression models. Age: adj BMI + sex is adjusted for BMI and sex, Age: adj BMI + sex + BCPs is additionally adjusted for BCPs. $\beta_{\Delta Ct}$: effect size, calculated on ΔCt values, (small ΔCt values indicate high miRNA levels), $\beta_{\Delta Ct} < 0$, positive association, implies higher miRNA levels with increasing age, $\beta_{\Delta Ct} > 0$ – negative association, indicate smaller miRNA levels with increasing age. *q*: corrected *p*-value using Benjamini-Hochberg multiple test correction. Accession number and additional information (gene family, MIPF gene family number, location on chromosome, strand) were derived from miRBase 21 (www.mirbase.org).
