# Supplementary material for: Associations of circulating plasma microRNAs with age, body mass index and sex in a population-based study
Source: BMC Med Genomics. 2015 Oct 14;8:61. doi: 10.1186/s12920-015-0136-7 (PMC4604724; doi:10.1186/s12920-015-0136-7)
Supplement: Additional file 3: Table S3. — Significantly BMI-associated miRNAs in different models. (PDF 196 kb) [file 12920_2015_136_MOESM3_ESM.pdf]

Additional file 3: Table S3. Significantly BMI-associated miRNAs in different models.

miRNA	BMI: adj age + sex (15) $\beta_{\Delta Ct}$	*q*	BMI: adj age + sex + BCPs (19) $\beta_{\Delta Ct}$	*q*	N	Accession	gene family	MIPF gene family	Chromo-some	strand
hsa-miR-106a-5p	0.02	1.99E-02	0.02	5.82E-03	366	MI0000113	mir-17	MIPF0000001	chrX	-
hsa-miR-122-5p	-0.08	2.61E-05	-0.08	2.25E-05	369	MI0000442	mir-122	MIPF0000095	chr18	+
hsa-miR-143-3p	0.04	2.61E-03	0.04	2.82E-03	314	MI0000459	mir-143	MIPF0000094	chr5	+
hsa-miR-145-5p	0.03	4.72E-03	0.03	1.13E-02	342	MI0000461	mir-145	MIPF0000079	chr5	+
hsa-miR-148a-3p	-0.04	2.61E-05	-0.04	2.25E-05	368	MI0000253	mir-148	MIPF0000056	chr7	-
hsa-miR-185-5p	0.02	1.32E-02	0.03	5.82E-03	370	MI0000482	mir-185	MIPF0000202	chr22	+
hsa-miR-193b-3p	-0.05	1.99E-02	-0.05	6.21E-03	208	MI0003137	mir-193	MIPF0000082	chr16	+
hsa-miR-194-5p	-0.03	4.72E-03	-0.04	1.68E-03	362	MI0000488	mir-194	MIPF0000055	chr1	-
hsa-miR-19b-3p			0.01	2.76E-02	368	MI0000074	mir-19	MIPF0000011	chr13	+
hsa-miR-20a-5p	0.02	3.16E-02	0.02	8.05E-03	367	MI0000076	mir-17	MIPF0000001	chr13	+
hsa-miR-215	-0.03	1.99E-02	-0.03	3.50E-02	363	MI0000291	mir-192	MIPF0000063	chr1	-
hsa-miR-365a-3p	-0.05	2.61E-03	-0.06	1.63E-03	258	MI0000767	mir-365	MIPF0000061	chr16	+
hsa-miR-486-5p			0.02	3.44E-02	368	MI0002470	mir-486	MIPF0000220	chr8	-
hsa-miR-505-3p	-0.05	1.02E-04	-0.06	3.62E-05	309	MI0003190	mir-505	MIPF0000217	chrX	-
hsa-miR-885-5p	-0.05	1.99E-02	-0.05	5.82E-03	315	MI0005560	mir-885	MIPF0000532	chr3	-
hsa-miR-93-5p	0.02	1.10E-02	0.02	3.21E-03	367	MI0000095	mir-17	MIPF0000001	chr7	-
hsa-miR-99a-5p	-0.04	3.24E-03	-0.04	2.63E-04	355	MI0000101	mir-10	MIPF0000033	chr21	+
hsa-miR-18a-5p			0.03	3.44E-02	351	MI0000072	mir-18	MIPF0000001	chr13	+
hsa-let-7i-5p			0.02	4.73E-02	367	MI0000434	let-7	MIPF0000002	chr12	+

BMI-associated miRNAs in different linear regression models. BMI: adj age + sex is adjusted for age and sex, BMI: adj age + sex + BCPs is additionally adjusted for BCPs. $\beta_{\Delta Ct}$: effect size, calculated on ΔCt values, (small ΔCt values indicate high miRNA levels), $\beta_{\Delta Ct} < 0$, positive association, implies higher miRNA levels with increasing BMI, $\beta_{\Delta Ct} > 0$ – negative association, indicate smaller miRNA levels with increasing BMI. *q*: corrected *p*-value using Benjamini-Hochberg multiple test correction. Accession number and additional information (gene family, MIPF gene family number, location on chromosome, strand) were derived from miRBase 21 (www.mirbase.org).
