# Supplementary material for: Associations of circulating plasma microRNAs with age, body mass index and sex in a population-based study
Source: BMC Med Genomics. 2015 Oct 14;8:61. doi: 10.1186/s12920-015-0136-7 (PMC4604724; doi:10.1186/s12920-015-0136-7)
Supplement: Additional file 4: Table S4. — Significantly sex-associated miRNAs in different models. (PDF 227 kb) [file 12920_2015_136_MOESM4_ESM.pdf]

Additional file 4: Table S4. Significantly Sex-associated miRNAs in different models.

	Sex: adj BMI + age (35)		Sex: adj BMI + age + BCPs (7)								
miRNA	β	*q*	β	*q*	N	Accession	gene family	MIPF gene family	Chromo-some	strand	potential miRNA origin
hsa-miR-145-5p	-0.39	3.95E-05	-0.35	5.42E-03	342	MI0000461	mir-145	MIPF0000079	chr5	+	PBMC[2]
hsa-miR-425-5p	0.23	2.62E-05			367	MI0001448	mir-425	MIPF0000242	chr3	-	
hsa-miR-32-5p	0.36	1.31E-04			364	MI0000090	mir-32	MIPF0000069	chr9	-	
hsa-miR-451a	0.31	2.16E-04			368	MI0001729	mir-451	MIPF0000148	chr17	-	E[1], H[4]
hsa-miR-22-3p	0.25	2.27E-04	0.26	5.42E-03	366	MI0000078	mir-22	MIPF0000053	chr17	-	
hsa-miR-16-2-3p	0.37	2.27E-04			353	MI0000115	mir-15	MIPF0000006	chr3	+	PBMC, H[4]
hsa-miR-142-3p	-0.27	2.27E-04	-0.27	5.42E-03	369	MI0000458	mir-142	MIPF0000084	chr17	-	G[1],T[1],PBMC[1]
hsa-miR-101-3p	0.19	3.57E-04			370	MI0000103	mir-101	MIPF0000046	chr1	-	
hsa-miR-144-3p	0.28	6.11E-04			369	MI0000460	mir-144	MIPF0000093	chr17	-	
hsa-miR-363-3p	0.26	1.14E-03			368	MI0000764	mir-92	MIPF0000138	chrX	-	
hsa-miR-15a-5p	0.18	1.14E-03			368	MI0000069	mir-15	MIPF0000006	chr13	-	
hsa-miR-19b-3p	0.17	1.14E-03			368	MI0000074	mir-19	MIPF0000011	chr13	+	
hsa-miR-29b-3p	0.23	1.45E-03			345	MI0000105	mir-29	MIPF0000009	chr7	-	
hsa-miR-424-5p	0.27	2.02E-03	0.33	5.42E-03	362	MI0001446	mir-322	MIPF0000164	chrX	-	MD[3]
hsa-miR-28-5p	-0.35	6.78E-03			277	MI0000086	mir-28	MIPF0000057	chr3	+	
hsa-miR-106b-5p	0.16	3.18E-03			369	MI0000734	mir-17	MIPF0000001	chr7	-	
hsa-miR-148a-3p	0.21	7.24E-03	0.27	1.29E-02	368	MI0000253	mir-148	MIPF0000056	chr7	-	
hsa-miR-143-3p	-0.28	1.23E-02			314	MI0000459	mir-143	MIPF0000094	chr5	+	PBMC[2]
hsa-miR-29c-3p	0.17	5.84E-03			367	MI0000735	mir-29	MIPF0000009	chr1	-	
hsa-miR-25-3p	0.19	6.78E-03			370	MI0000082	mir-25	MIPF0000013	chr7	-	
hsa-miR-19a-3p	0.14	7.24E-03			369	MI0000073	mir-19	MIPF0000011	chr13	+	
hsa-miR-26a-5p	-0.20	1.10E-02			369	MI0000083	mir-26	MIPF0000043	chr3	+	G[1],PBMC[1]
hsa-miR-150-5p	-0.22	7.29E-03	-0.31	5.42E-03	369	MI0000479	mir-150	MIPF0000197	chr19	-	PBMC, H[4]
hsa-miR-16-5p	0.16	1.23E-02			369	MI0000070	mir-15	MIPF0000006	chr13	-	
hsa-miR-205-5p	-0.44	1.48E-02			135	MI0000285	mir-205	MIPF0000058	chr1	+	
hsa-miR-485-3p	-0.24	2.10E-02			342	MI0002469	mir-485	MIPF0000201	chr14	+	
hsa-let-7d-5p	-0.22	2.62E-02			358	MI0000065	let-7	MIPF0000002	chr9	+	T[1]
hsa-miR-766-3p	-0.28	2.39E-02			240	MI0003836	mir-766	MIPF0000677	chrX	-	
hsa-miR-501-3p	-0.30	2.68E-02			216	MI0003185	mir-500	MIPF0000139	chrX	+	
hsa-miR-130a-3p	0.19	2.11E-02			367	MI0000448	mir-130	MIPF0000034	chr11	+	

	Sex: adj BMI + age (35)		Sex: adj BMI + age + BCPs (7)								
miRNA	β	*q*	β	*q*	N	Accession	gene family	MIPF gene family	Chromo-some	strand	potential miRNA origin
hsa-miR-146b-5p	-0.27	2.11E-02			138	MI0003129	mir-146	MIPF0000103	chr10	+	
hsa-miR-342-3p	-0.18	2.62E-02			369	MI0000805	mir-342	MIPF0000190	chr14	+	
hsa-let-7c					317	MI0000064	let-7	MIPF0000002	chr21	+	
hsa-miR-195-5p	-0.32	3.62E-02			146	MI0000489	mir-15	MIPF0000006	chr17	-	
hsa-miR-301a-3p					294	MI0000745	mir-130	MIPF0000034	chr17	-	
hsa-miR-92a-3p	0.11	4.92E-02			369	MI0000093	mir-25	MIPF0000013	chr13	+	
hsa-miR-30d-5p	0.18	3.72E-02	0.33	5.42E-03	327	MI0000255	mir-30	MIPF0000005	chr8	-	

Sex-associated miRNAs in different linear regression models. Sex: adj BMI + age is adjusted for BMI and age, Sex: adj BMI + age + BCPs is additionally adjusted for BCPs.

$\beta_{\Delta Ct}$: effect size, calculated on ΔCt values, (small ΔCt values indicate high miRNA levels), $\beta_{\Delta Ct} < 0$, positive association, implies a female specific miRNA, $\beta_{\Delta Ct} > 0$ – negative association, indicates a male specific miRNA. *q*: corrected *p*-value using Benjamini-Hochberg multiple test correction. Accession number and additional information (gene family, MIPF gene family number, location on chromosome, strand) were derived from miRBase 21 (www.mirbase.org).

G: granulocytes (basophils, eosinophils, neutrophils), E: erythrocytes, T: thrombocytes, PBMC: peripheral blood mononucelar cells (monocytes: macrophages, dendritic cells; lymphocytes: B-cells, T-cells, NK-cells), MD: monocyte differentiation, H: hematopoiesis;

references: [1] Teruel-Montoya R, Kong X, Abraham S, Ma L, Kunapuli SP, Holinstat M, et al. MicroRNA expression differences in human hematopoietic cell lineages enable regulated transgene expression. PLoS One. 2014; 9: e102259.; [2] Zhou J, Chaudry H, Zhong Y, Mustafa AM, Perkins LA, Owens WB, et al. Dysregulation in microRNA expression in peripheral blood mononuclear cells of sepsis patients is associated with immunopathology. Cytokine 2015; 71: 89-100.; [3] Dai R, Ahmed SA Sexual dimorphism of miRNA expression: a new perspective in understanding the sex bias of autoimmune diseases. Ther Clin Risk Manag. 2014; 10: 151-163.; [4] Zimmerman AL, Wu S MicroRNAs, cancer and cancer stem cells. Cancer Lett. 2011; 300: 10-19.
